# A Comparison of implant impression precision: Different materials and techniques

**DOI:** 10.4317/jced.54457

**Published:** 2018-02-01

**Authors:** Mahtab Tabesh, Marzieh Alikhasi, Hakimeh Siadat

**Affiliations:** 1 DDS. Dentist, Dental Research Center, School of Dentistry, Tehran University of Medical Sciences, Tehran, Iran; 2 DDS, MSc. Associate Professor, Dental Research Center, Dental Implant Research Center, Dentistry Research Institute, Department of Prosthodontics, School of Dentistry, Tehran University of Medical Sciences, Tehran , Iran; 3 DDS, MSc. Professor, Dental Research Center, Dental Implant Research Center, Dentistry Research Institute, Department of Prosthodontics, School of Dentistry, Tehran University of Medical Sciences, Tehran , Iran

## Abstract

**Background:**

Precision of implant impressions is a prerequisite for long-term success of implant supported prostheses. Impression materials and impression techniques are two important factors that impression precision relies on.

**Material and Methods:**

A model of edentulous maxilla containing four implants inserted by All-on-4 guide was constructed. Seventy two impressions using polyether (PE), polyvinyl siloxane (PVS), and vinyl siloxanether (VSE) materials with direct and indirect techniques were made (n=12). Coordinates of implants in casts were measured using coordinate measuring machine (CMM). Data were analyzed with ANOVA; t-test and Tukey test were used for post hoc.

**Results:**

With two-way ANOVA, mean values of linear displacements of implants were significantly different among materials and techniques. One-way ANOVA and Tukey showed significant difference between PE and VSE (*P*=0.019), PE and PVS (*P*=0.002) in direct technique, and between PVS and PE (*P*<0.001), PVS and VSE (*P*<0.001) in indirect technique. One-way ANOVA and t-test showed significant difference between the two techniques in PVS groups (*P*<0.001) and in PE groups (*P*=0.02). Two-way ANOVA showed mean values of rotational displacement of implants were significantly different among materials. One-way ANOVA and Tukey test showed significant difference between PVS and PE (*P*=0.001) and between PVS and VSE (*P*=0.012) in indirect groups.

**Conclusions:**

On the basis of the results, when deciding on the material to make an impression of implants, PE is recommended for direct technique while PE and VSE are recommended for indirect technique. Recommended technique for VSE is either direct or indirect; and for PE and PVS is direct.

** Key words:**Polyvinyl siloxane, polyether, vinyl siloxanether, direct technique, indirect technique, All-on-4, coordinate measuring machine.

## Introduction

Today dental implants are widely used in oral rehabilitation of patients and their longitudinal effectiveness has been proved by many clinical studies (1). Endosseous implants lack periodontal ligament support and cushion effect to compensate for stresses caused by inaccuracies in dental prosthesis. Lack of passivity may lead to both biological and mechanical complications such as screw loosening, fracture of implant components and occlusal inaccuracy. Therefore, fabrication of superstructure must ensure the most attainable passive fit (2-4). The first and the most crucial step to achieve passive fit is making an accurate impression which precisely transfers interimplant dimensions (4-7). Precision of implant impressions are influenced by many factors including impression material, impression technique, splinting of impression copings, level of impression and depth and angulation of implants (2,5,6,8-10).

A variety of impression materials have been suggested for implant impressions such as impression plaster, hydrocolloids and elastomers with four basic types of polysulfides, polyether, condensation silicones and polyvinyl siloxane which is also known as addition silicones (11). Polysulfides show good reproduction of surface details; however they are not dimensionally stable if stored for longer period of time (12). Significant disadvantage of condensation silicones is their shrinkage due to evaporation of volatile by products released in polymerization reactions (13). Property of impression material to prevent positional distortion between implant analogues caused by accidental displacement of impression copings is a key factor; therefore polyvinyl siloxane and polyether have been suggested as materials of choice (6,8,14,15). Polyether has been recommended for implant impressions because of its dimensional stability, rigidity, tear resistance and hydrophilicity (4,5,14). Polyether chemical structure contains carbonyl and ether functional groups which allow water molecules to interact through hydrogen bonding; therefore if stored in contact with moisture, it may encounter swelling with an accompanying loss of accuracy (7,13). The other material frequently used is polyvinyl siloxane which shows many desirable properties of polyether respecting the quality of implant impressions, at a lower cost (4). It has been reported that putty and light-body combination of polyvinyl siloxane results in more precision than medium-body polyether when implants are located deep subgingivally (2). Comparing these two materials, some studies advocate polyvinyl siloxane, (13,16) some advocate polyether (17) while others found no significant difference (5,8,18)

Development of material science has led to incorporating qualities of polyether and polyvinyl siloxane into a newer vinyl siloxanether material (12,15,19,20) Vinyl siloxanether has been claimed by the manufacturer to possess good mechanical and flow properties on top of excellent wetting characteristics in both unset and set conditions (11,15). The other advantage of vinyl siloxanether is that it achieves its final hardness immediately after setting (11). Moreover, creating a chemical bond between vinyl siloxanether and polyvinyl siloxane is possible (11). Yet, the precision of this newly formulated material has to be established (15).

Different techniques of impression making can also influence the precision. Two impression techniques of direct and indirect are commonly used to transfer the intraoral position of implants to working casts. There are numerous studies suggesting that direct impression technique leads to more precision, (3,11,21-23) while some other investigations favor the indirect technique (2,3,8,14,24). It has been proposed to use direct technique with multiple angulated implants while indirect technique can be employed in parallel or divergent, 2-implant situation (4). In addition, most clinicians find the indirect technique less challenging especially when implants are positioned in posterior region or patients show excessive tendency for gag reflex or when intermaxillary distance is inadequate in opening (5). Moreover, digital dentistry has introduced new methods in which conventional impression materials and technique are substituted with intra-oral scanners and related systems. These new technologies are finding their way into procedures related to fabrication of implant prostheses as well. The purpose of this in vitro study was to compare polyvinyl siloxane, polyether and vinyl siloxanether impressions with two techniques of direct and indirect impression making in terms of precision.

## Material and Methods

**Figure 1:**
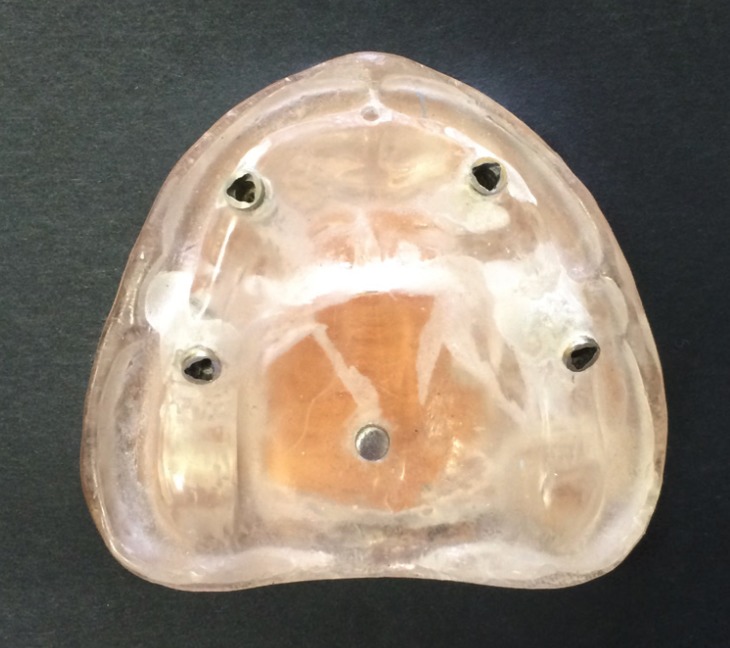
The master model with four implants inserted by All-on-4 guide.

A master model of edentulous maxilla was constructed using autopolymerizing acrylic resin (Technovis 4000, Heraeus Kulzer GmbH & Co, Wehrheim, Germany). Four implants (Replace select; Noble Biocare, Goteborg, Sweden) of 4.3 mm diameter and 11 mm length were inserted in bilateral canines and second premolars sites according to All-on-4 protocol. Anterior two implants were parallel to each other and perpendicular to edentulous ridge while posterior ones were tilted 45° distally. A metal cylinder was placed perpendicular to model plane in posterior part of midpalatal raphe and was served as the reference point in measurements and its top surface was considered to be the horizontal plane (Fig. 1). Linear and angular positions of implants were then measured and registered as baseline data using CMM (Coordinate Measuring Machine, Mistral; DEA Brown & Sharpe, Grugliasco, Italy).

In this study, three impression materials and two different impression techniques were used. Impression materials included medium-body polyvinyl siloxane (Zhermack Elite HD+ Regular Body, Kouigo, Italy), medium-body polyether (Impregum Soft; 3M ESPE, Seefeld, Germany) and medium consistency vinyl siloxanether (EXA’lence; GC Corporation, Tokyo, Japan). Two techniques of direct (pick-up) and indirect (transfer) were used. A total number of 72 impressions were made, 12 for each group. Fabrication of custom trays were done by fastening closed tray impression copings on master model, then blocking the impression copings out by adapting base-plate wax (Modeling wax; Dentsply, Weybridge, UK) around and over them. An impression of irreversible hydrocolloid (Chromogel; Marlic Medical Industries Co, Tehran, Iran) was taken and the resultant cast was fabricated using dental stone type IV (GC FUJIROCK EP; GC America Inc, Illinois, America) and autopolimerizing acrylic resin (Duralay; Reliance Dental Mfg. Co, Illinois, America) in positions of impression copings. The obtained cast was used to fabricate color-coded custom trays. The cast was covered by one layer of 2mm base-plate wax to allow a reliable thickness of impression material. To ensure standard positioning of tray during impression making, three tissue stops were included in each tray. Seventy two 2mm-thick custom trays (36 open and 36 closed trays) were fabricated using light polymerizing acrylic resin sheets (Megatray; Megadenta, Radebery, Germany), twelve for each group. Trays were perforated in distances of one cm and proper tray adhesives were applied inside and 5 mm around tray periphery 15 minutes prior to impression makings. Impression materials were mixed according to manufacturers’ instructions and an amount of them were placed around and over impression copings before carrying the material with tray to ensure complete coverage of them.

**Figure 2:**
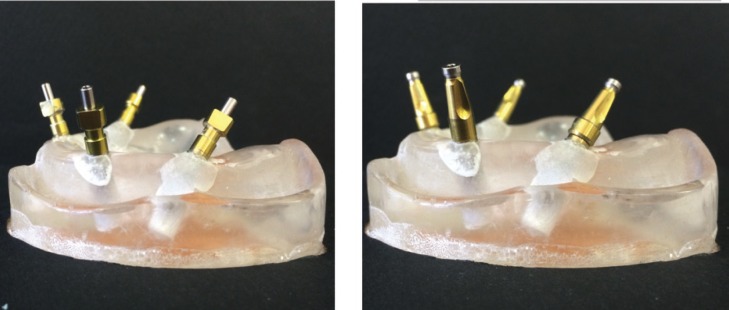
Impression copings secured in place for indirect (left) and direct (right) techniques.

In order to make indirect impressions, closed impression copings were secured at 10 Ncm torque on implants. Direct impressions were made using open impression copings secured with the same torque (Fig. 2). After placing some impression material around impression copings, impression trays were filled and seated in place with a 5 kg weight placed on the tray to standardize seating load for each impression. All impressions were made in a temperature-controlled environment and allowed to set in distilled water at 37±2°C to stimulate intraoral situation. Once the impression material was set, for indirect impressions, the tray was removed, implant analogues were attached to copings and the impression coping-analogue assembly was reinserted into the impression in respective notch left in the impression, by firmly pushing it into place and slightly rotating to feel for the anti-rotational resistance. For direct impressions, the impression coping screw was unfastened and the tray was removed with impression copings buried in the impression. The implant analogues were attached to the copings. Afterwards, impressions were poured with dental stone type IV (GC FUJIROCK EP) with a powder/water ratio of 30g/7ml. Two hours later the impression was separated from the cast and casts were coded for measurements. All laboratory procedures were done by the same operator.

**Figure 3:**
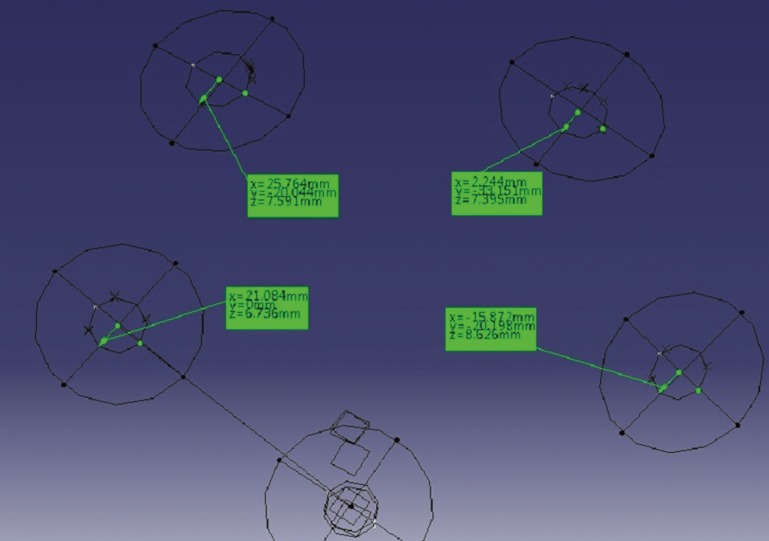
Coordinates of implant heads measured by CMM.

All measurements were performed by one calibrated, blinded examiner. Each cast was measured three times and an average was calculated. For each implant on the casts, the x, y and z coordinates were measured by recording the distances from the reference point on the center of the superior surface of the reference cylinder to center of the implant in each dimension (Fig. 3). The x axis was defined as the line connecting the reference point to center of the implant, while the y axis was defined as the perpendicular line to the x axis in the horizontal plane and the z axis was defined as the perpendicular line to the x axis in the vertical plane. To evaluate angular changes (∆θ), one flat side of closed impression copings placed on the implants was used for measuring the rotation. To represent three dimensional displacements, Euclidian distance of implants from reference point was calculated using ∆r^2=∆x^2+∆y^2+∆z^2 formula. Data was analyzed with two-way ANOVA followed by one-way ANOVA, Tukey, and t-test tests. Significance level was set at 0.05.

## Results

-Linear Displacement

Absolute mean values and standard deviations of implant head displacements in x, y and z coordinates and total linear displacements (r) are presented in  Table 1. Two-way ANOVA showed that impression material and technique both had significant effect on the precision (*P*<0.001). One-way ANOVA and Tukey test, showed direct technique with polyether more precise than both vinyl siloxanether and polyvinyl siloxane (*P*=0.019 and *P*=0.002 respectively), while no significant difference was seen between vinyl siloxanether and polyvinyl siloxane. The same test showed indirect technique with polyvinyl siloxane less precise compared to polyether (*P*<0.001) or vinyl siloxanether (*P*<0.001), however the results from polyether and vinyl siloxanether did not differ significantly. One-way ANOVA and t-test showed direct technique to be more precise in polyvinyl siloxane (*P*<0.001) and polyether (*P*=0.001) groups, but in vinyl siloxanether group, selection of technique did not affect precision significantly.

**Table 1:**
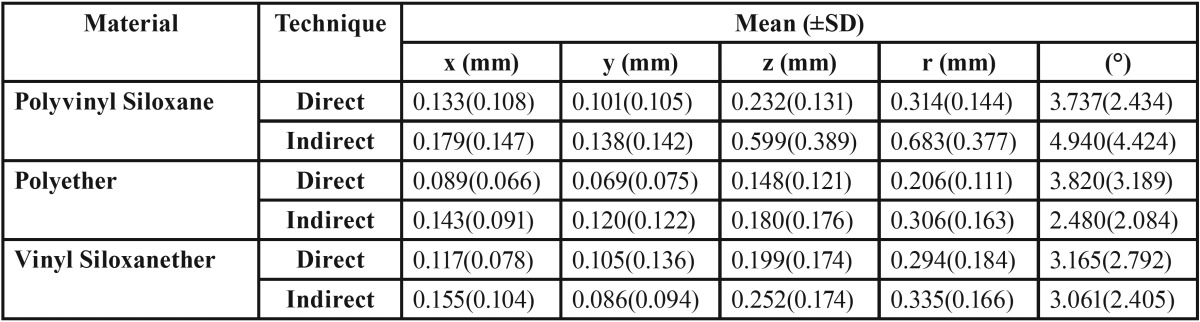
Mean values and standard deviations of linear and rotational displacement of implant head in casts.

-Rotational Displacement

Absolute mean values and standard deviations of implant head rotational displacements (θ) are also presented in  Table 1. Two-way ANOVA showed no significant difference between impression techniques, while significant difference was observed between impression materials. With One-way ANOVA it was shown that when using an indirect technique both polyether and vinyl siloxanether cause less displacement than polyvinyl siloxane (*P*=0.001 and *P*=0.012 respectively). No significant difference was found between other study groups.

## Discussion

There have been numerous attempts towards eliminating clinical complications of dental implants and their relying prostheses by making the clinical and laboratory procedures as precise as possible. These include exploiting different impression materials characteristics to best record intraoral implants situations as well as innovating different methods of impression making using different designs of available implant components (2). In the present study the effect of different impression materials, polyether, polyvinyl siloxane and vinyl siloxanether besides the effect of impression techniques, direct and indirect, and their interactions on the precision of obtained casts were investigated.

Comparing the three materials, polyether showed more precision than polyvinyl siloxane and vinyl siloxanether in linear transfer of implants locations when using the direct technique. However, if the indirect technique was used polyether and vinyl siloxanether were more precise than polyvinyl siloxane. More precision obtained from polyether impressions may be contributed to its high rigidity which helps holding the impression copings in their position while removing the impression and therefore, minimal positional displacement of impression copings occurs. These findings are consistent with Kankane’s study which examined polyether and polyvinyl siloxane impression precision of a master model of an edentulous mandible containing four implants (25). In another study about parallel and angulated implants, it was shown that polyether and polyvinyl siloxane result in more precision than vinyl siloxanether (26). In a study by Vojdani *et al.* no significant difference was seen between polyether, polyvinyl siloxane and vinyl siloxanether in parallel implants while polyvinyl siloxane showed better results than vinyl siloxanether and vinyl siloxanether showed better results than polyether in angulated implants. There are also studies that favor usage of vinyl siloxanether as impression material instead of polyether or polyvinyl siloxane which are mainly related to making impression of prepared teeth in fixed prosthodontics rather than implants (7,12). One reason may be the higher tensile strength of vinyl siloxanether compared to the two other materials, which enhances recording the interproximal and crevicular areas in which impression material has little thickness. Considering the fact that implant impressions lack such critical araes, higher tensile strength would not be such a privilege. Studies by Reddy, Seyedan and Karl showed little or no difference between polyether and polyvinyl siloxane when using a direct technique (5,6). Some other studies (27,28) reported polyvinyl siloxane to be more precise than polyether with direct technique and another (14) study favored polyvinyl siloxane with indirect technique. Considering rotational transfer precision of implants in the present study, it was conceived that with an indirect impression technique, polyether and vinyl siloxanether result in less discrepancy compared to polyvinyl siloxane. Wee’s study also showed better results with polyether rather than polyvinyl siloxane but with a direct impression technique (8). In contrast, Lorenzoni *et al.* reported less rotational displacement with polyvinyl siloxane and indirect technique (14).

As it can be seen, literature is inconsistent regarding impression materials precision. This is mostly due to different methodology of these studies. Definition of precision, devices used to measure the discrepancies between the original model and the duplicates, parameters that were evaluated and sample sizes of the studies were not all the same. Number of the present implants, system of implants and manufacturers of materials were among a wide range. Even within a specific type of material, different viscosities of the same impression material show different mechanical properties, hence their ability to withstand stresses before a permanent deformation occurs would be different (29).

The obtained results can be partly explained through different inherent characteristics of each material. Polyether is best known for its rigidity which helps keeping impression copings firmly in place to ensure least accidental rotation of impression copings while fastening the screws (26). This ability becomes more highlight when a direct technique is employed to multiple parallel implants. On the other hand, flexibility accompanying elastic recovery becomes a more important factor especially if implants are angulated, because with angulated implants more stress will be loaded on impression material upon removal. This gives polyvinyl siloxane with its high elastic recovery a privilege over others (26).

Literature has also been in favor of using vinyl siloxanether, mainly for prepared teeth in fixed prosthodontics. There are several critical areas when making an impression of a prepared tooth. One is the finishing line especially those which are located subgingivally. Vinyl siloxanether has a better tensile strength compared to polyether or polyvinyl siloxane (29). This property along with high flowability makes it possible to record narrow crevicular areas and finishing lines. Since there is no specific finishing line for implant impressions, this property does not seem to improve precision of implant impression.

Comparing the two different techniques used in this study, direct impression technique significantly resulted in more precision of linear transfer of implants when using polyvinyl siloxane or polyether. The highest values of discrepancy in indirect technique were found in the z axis which might be the result of an incorrect repositioning of the impression copings. Indirect technique also resulted in less rotational discrepancy when using polyether. No significant difference was seen between the two techniques with vinyl siloxanether in either linear or rotational transfer of implants locations. Literature mostly shows no difference or better results with direct technique (2,3,6,30). However, there are studies that favor indirect technique for example Balouch *et al.* found indirect technique to be more precise which they explained to be a result of technique simplicity (31). Moreover using direct technique in some clinical cases for example when there is not enough intra arch space or when the patient shows exaggerated gag reflex can impose major difficulties in impression making (32). Therefore, other factors besides precision must be taken into account when selecting the technique in some cases.

One important point that needs attention when interpreting studies is machining tolerance among different implant components (26). Machining tolerance is the probable mismatch of paired machined components and is reported to range from 22 to 100 µm (33). This means regardless of other factors affecting the precision of implant impressions, an inaccuracy among this range can always occur and should not be misinterpreted.

There are as well some limitations in this *in vitro* study. The master model used in the study was made of hard, rigid acrylic resin while intra oral soft tissues are flexible and tend to distort when different forces are applied. Presence of saliva, blood and other biological secretions will also affect precision of impressions. To validate this study, long-term *in vivo* studies would have to be done in future.

## Conclusions

Within limitations of this study, it was concluded that all the three impression materials tested in this study may lead to some extent of discrepancy. If a direct technique is considered polyether is the better choice, while for indirect technique polyether and vinyl siloxanether are choices. If vinyl siloxanether is the material, then both direct and indirect techniques are favorable, though if polyvinyl siloxane or polyether is the material, less displacement of implants will be achieved using a direct technique.
